# Screening of Dementia in Portuguese Primary Care: Methodology, Assessment Tools, and Main Results

**DOI:** 10.3389/fmed.2017.00197

**Published:** 2017-11-13

**Authors:** Laetitia Teixeira, Pedro Machado Dos Santos, Sara Alves, Maria João Azevedo, Mafalda Gomes Duarte, António Leuschner, Constança Paúl

**Affiliations:** ^1^UNIFAI, Instituto de Ciências Biomédicas Abel Salazar, Universidade do Porto, Porto, Portugal; ^2^CINTESIS, Instituto de Ciências Biomédicas Abel Salazar, Universidade do Porto, Porto, Portugal; ^3^EPIUnit, Instituto de Saúde Pública, Universidade do Porto, Porto, Portugal; ^4^Hospital Magalhães, Porto, Portugal

**Keywords:** caregivers, cognitive decline, dementia, old people, primary care

## Abstract

The objectives of this article are as follows: (1) to describe the assessment protocol used to outline people with probable dementia in Primary Health Care; (2) to show the methodological design and procedure to obtain a representative sample of patients with probable dementia; and (3) to report the main characteristics of the sample collected in the context of the study “Characteristics and needs of people with probable dementia.” The study protocol was based on the “Community Assessment of Risk and Treatment Strategies (CARTS) Program” and is composed by a set of instruments that allow the assessment of older adults with probable dementia in several areas (health, psychological, functionality, and other). Descriptive analysis was used to characterize the final sample (*n* = 436). The study protocol as well as the methodological procedure to obtain the referral of research participants and data collection on the condition of people with probable dementia in Primary Health Care proved to be a valuable tool to obtain a sample of patients distributed by the full range of probable dementia in a large geographical area. Results may allocate the design of care pathways for old people with cognitive disorders to prevent, delay impairment, and/or optimize quality of life of patients.

## Introduction

The Portuguese Census 2011 (1) showed that in Portugal 19.1% of the total population (*n* = 2,010,064) was aged 65 or plus. According some projections, this population will increase, with the group age 80 + reaching the 15% in 2060 (2).

Presently little information is known in Portugal about the needs of the old people with probable dementia and their informal caregivers. Nunes et al. (3) estimated that the prevalence of cognitive impairment and dementia in Portuguese people living in the north of the country were 16.8 and 2.1% in rural areas, and 12 and 2.7% in urban areas, respectively; the majority of the reported cases were related with cerebrovascular diseases and vascular risk factors (48%). A more recent study (4) revealed that the prevalence of dementia/Alzheimer’s disease was 5.91% in the Portuguese population with 60 or more years old. Considering the international context, the prevalence of dementia estimated by the World Health Organization (WHO) in South Europe increases with age, varying between 0.026% for individuals aged 65–69 years and 0.324% for those aged 85+ years (5). In addition, it will be expected an increase in the number of people with dementia, doubling by 2030 and tripling by 2050. These numbers have a high impact in the quality of life of the people and in the economy of the families and communities, representing one of the highest challenges/priorities of public health offices/professionals.

Early detection of probable dementia is very important, and it appears to be under diagnosed by general practitioners (GPs). Nevertheless, GPs are well positioned to notice the possible cognitive decline of their patients and can be a major potential source for increasing the rate of case detection [e.g., Ref. (6, 7)]. In a Finnish population based study, Lopponen et al. (8) found less than 50% of the patients with dementia with a diagnosis documented in primary care; the existence of diagnosis increased in more advanced stages of dementia.

High levels of poverty need to be considered in the topic of dementia (9–11) and must be addressed together with cultural aspects in the Portuguese context, namely, the low educational levels. This cross-sectional study has a main objective to draw a physical and mental health profile of the old people with dementia living in the north of Portugal and to understand their risk situation to further planning adequate responses and services for this specific population.

The main objectives of this article are as follows: (1) to describe the assessment protocol used to outline people with probable dementia in Primary Health Care; (2) to show the methodological design and procedure to obtain a representative sample of patients with probable dementia; and (3) to report the main characteristics of the sample collected in the context of the study “Characteristics and needs of people with probable dementia.”

## Materials and Methods

### Participants

The population of this study was defined as Portuguese people with 65 years and over, living in the community in the geographical area covered by the Portuguese North Regional Health Authority (ARS North) with mental health concerns. The geographical area is composed by 86 municipalities, which are organized in 24 Associations of Health Centres (ACES). The inclusion criteria were as follows: (a) outpatient of a health-care units integrated in an ACES covered by the ARS North and (b) age 65 or + years old. The exclusion criteria were as follows: (a) patient not using a primary health-care unit covered by the ARS North; (b) age less than 65 years old; (c) living in nursing home, hospital or psychiatric institution; and (d) absence of memory concerns [patients classified in stage 1 of the Global Deterioration Scale (GDS) (12, 13)].

### Sample

Based on the distribution of Portuguese population with 65+ years old (1) and on the prevalence of dementia in the Western Europe predicted by the WHO (5), an estimate of Portuguese population with dementia by age groups is presented in Table 1. The sample size, calculated for each age group, was considered as 1% of the estimated population with dementia. 572 participants with probable dementia compose the final sample. Table 1 the sample size calculation (total and by age group) according the prevalence of dementia.

**Table 1:** Sample size calculation (total and by age group) according the prevalence of dementia.

	65–69 years	70–74 years	75–79 years	80–84 years	85+ years	Total
Population	180,352	150,687	136,275	97,113	72,399	636,826
Prevalence of dementia	0.026	0.043	0.074	0.129	0.324	–
Estimated population with dementia	4,689	6,480	10,084	12,528	23,457	57,238
Final sample (1%)	47	65	101	125	235	572

### Measures

The study protocol was based on the “Community Assessment of Risk and Treatment Strategies (CARTS) Program” developed in the University College Cork, Ireland (14). The study protocol includes instruments divided in three main parts: Part A: assessment of the patient with probable dementia; Part B: assessment of the patient with probable dementia by the health professional (GP or nurse); Part C: evaluation of the informal caregiver of the patient with probable dementia (if available). Table 2 resumes the domains evaluated and the instruments used in each part of the study protocol.

**Table 2:** Study protocol: domains and instruments used in each part.

Part A		
A1. Sociodemographic questionnaire	Sex	Infrastructures accessibilities
	Age	Formal care
	Education level	Informal care
	Profession	Use of health services
	Marital status	Medication
	Household	Health subsystem
	Residence context	Health expenditures
	Type of residence	

A2. Cognition	Mini-Mental State Examination (15, 16)	
	Global Deterioration Scale (12, 13)	

A3. Depression	AB Clinician Depression Screen (ABCDS) (17)	

A4. Biobehavioral aspects	Timed “Up and Go” (18)	
	Malnutrition Universal Screening Tool (19, 20)	
	Short-Form Mini-Nutritional Assessment (21, 22)	
	Bedside Swallow Assessment	
	Handgrip strength	
	Exhaustion	
	Physical activity	
	Tobacco and alcohol consumption	
	Whispered Voice Test (23)	
	Snellen Test (24)	

**Part B**		

B1. Physical health	Older Americans Resources and Services (25, 26)	

B2. Adverse events	The Community Assessment of Risk Tool (14)	

**Part C**		

C1. Caregiver burden	Caregiver Burden Score (27)	

C2. Depression	ABCDS (17)	

C3. Mental health	Neuropsychiatric Inventory Questionnaire (28, 29)	

*Mini-Mental State Examination* (15, 16) is widely used for cognitive decline screening and is composed by 19 questions divided in 6 domains. The final score vary between 0 and 30. *GDS* (12, 13) is used to classify individuals with cognitive decline according to a scale of seven points: 1. Without cognitive decline; 2. Very mild cognitive decline; 3. Mild cognitive decline; 4. Moderate cognitive decline; 5. Moderately severe cognitive decline; 6. Severe cognitive decline; and 7. Very severe cognitive decline. *AB Clinician Depression Screen* (17) is a brief version of the Geriatric Depression Scale and is composed by five dichotomist questions (yes/no). The final score vary between 0 and 5, and individuals with a score equal or higher to 3 present high probability of depression. *Timed “Up and Go”* (18) is a simple test used to assess a person’s mobility, using the time that a person takes to rise from a chair, walk 3 m, turn around, walk back to the chair, and sit down. *Malnutrition Universal Screening Tool* (19, 20) is a five-step screening tool to identify adults at risk of malnutrition or obese. The final score vary between 0 and 6, considering three categories: 0. Low risk; 1. Moderate risk; and ≥2. High risk. *Short-Form Mini Nutritional Assessment* (21, 22) is a valid nutrition screening and assessment tool that can identify patients who are malnourished or at risk of malnutrition and consist in six questions. The score vary between 0 and 14 and a score equal or higher to 11 is indicator of possible malnutrition. *Bedside Swallow Assessment* allows the evaluation of swallowing after sitting the people in a right posture and asking the person to drink 30 ml of water. Three criteria were recorded and the final score of the test corresponds to the number of observed criteria: 1. No criteria; 2. Presence of 1 criterion; 3. Presence of 2 or more criteria. *Handgrip strength* is evaluated using a dynamometer considering four attempts, two in each hand. The final score correspond to the mean of the highest values. *Exhaustion* is evaluation considering a dichotomy question (yes/no) “In the last month, do you feel that you had very little energy to do the things you wanted to do?” *Physical activity* frequency evaluated using a four-point question: 1. >1/week; 2. 1/week; 3. 1–3/month; and 4. Almost never or never. *Alcohol and tobacco* consumption evaluated considering a set of questions about quantity, duration, and type. *Whispered Voice Test* (23) evaluate the audition and *Snellen Test* (24) the vision The physical health dimension of the *Older Americans Resources and Services* (25, 26) comprises a checklist of 16 diagnoses. *The Community Assessment of Risk Tool-CART* (14) evaluates the perceived risk of three adverse events: institutionalization, hospitalization and death. *Caregiver Burden Score* (27) assess the caregiver burden and score vary between 0 and 30. Score equal or higher to 15 is indicator of burden. *Neuropsychiatric Inventory Questionnaire* (28, 29) is a brief version of the Neuropsychiatric Inventory and allows the evaluation of psychopathology in dementia and its repercussion on the caregiver’s overload. For each symptom, it evaluates the presence (yes/no), severity (1. Low; 2. Moderate; and 3. Severe) and caregiver distress (0. Not at all; 1. Minimally; 2. Mildly; 3. Moderately; 4. Severely; and 5. Very severely or extremely).

### Ethical Procedure

The study was submitted to the ethical committee of the ARS North—procedure number 6/2014 and approved at 7 January 2014. All the participants signed the informed consent form that was developed according the Declaration of Helsinki.

### Data Collection

**Figure 1:**
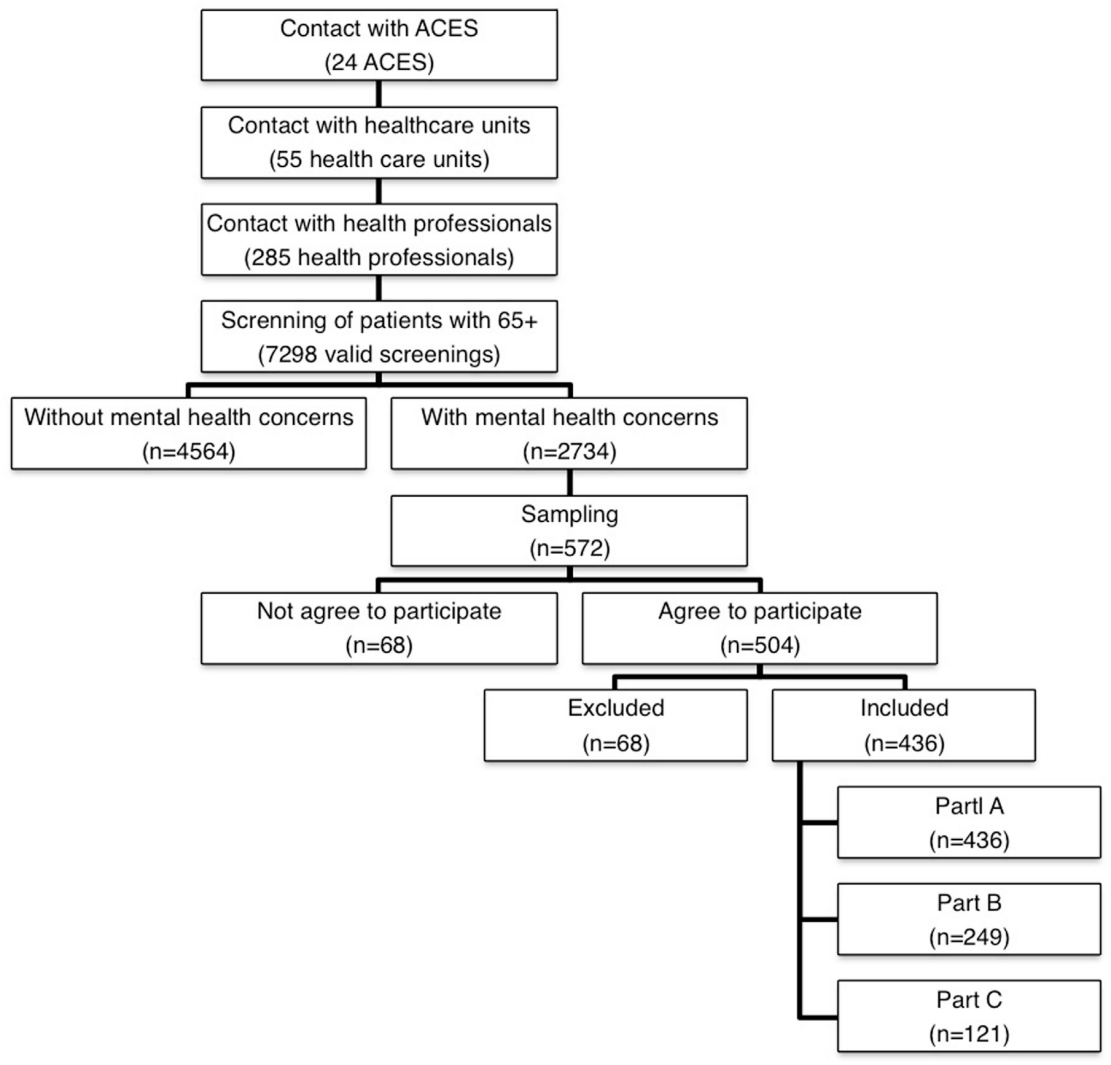
Data collection’s flowchart.

The data collection started in 2014 January and ended in 2016 April. Figure 1 shows the data collection’s flowchart.

The first step consisted in the contact with the 24 ACES to obtain the authorization to do the study. All ACES had accepted to participate.

The second step consisted in contacting at least two health-care units of each ACES with health professionals presenting interest in participating in the study. The health professional had as main responsibility filling in the screening instrument regarding the identification of people at risk of adverse health outcomes, namely, mental health concerns. The instrument used was the Risk Instrument for Screening in the Community (30), which is a new risk instrument for screening of old people. Based on the information about the patient, the health professional classified the patient in three different domains (mental health, ADLs, and physical/medical health) in a perceived risk scale (from 1. Minimum risk to 5. Extreme risk) for the following three adverse events: hospitalization, institutionalization, and death. All health professional involved in this step received training to use this instrument by the investigators and the sessions took place in the health-care unit facilities. In this screnning, 55 health-care units were enrolled, with the participation of 285 health professionals who filled 7,298 valid screenings.

In the third step, and based on this screening and considering only patients with mental health concerns (*n* = 2,734), the sample was calculated using the stratified probability sampling method, considering sex, age groups, and ACES as strata. The technique used to extract patients for the sample was the lottery technique. Each patient with mental health concerns received a random number. The patients who received the higher numbers were invited to participate in the study, until all planned quotas were completed.

The health-care office contacted the selected patients, explaining the purpose of the study; if the patients agreed to participate they were further referred to the research team. In a second moment, the interviewers contacted the patients to schedule the interviews according to their availability. A limit of four contacts was fixed until a patient was withdrawn. In these situations, if available, another patient with similar conditions of the previous one was selected, according the sampling method described earlier.

The majority of the interviews were done in the health-care units (79.6%), in an appropriate local where confidentiality was guaranteed. If it was impossible to do the interview in the health-care unit, the interviews were completed at patients’ home (19.9%). The main reason for the interviews to take place at home was the incapacity of the patient due to being bedridden or presenting low mobility. In the first moment of the interview, the patient was informed about the conditions of participation in the study, with the opportunity to clarify doubts. In order to formalize the interest of the patient in participating in the study, a personal Informative Consent was signed. If the patient did not have cognitive capacity, the signature of the consent was required to his/her legal representative.

The study protocol took on average 45 min to complete. If the informal caregiver was present, the interviewer asked him/her to fill the Part C. After the interview, the health professional (GP or nurse) complete the Part B.

Regular meetings occur between the interviewers and the coordinator of the study with the purpose of supervising and monitoring of the data collection. The planning of data collection, the discussion of cases and the analysis of problems related with the scoring of the scales included in the study protocol were the main aspects discussed in the meetings.

The final sample comprised 436 patients with probable dementia. The ratio of execution was 76.2%. The observed differences between the expected and collected samples are associated with some constrains related with the data collection, namely, difficulties/mistakes in the referral of cases and the high number of refuses to participate in the study (Figure 1).

### Statistical Analysis

Given the presence of non-response related with the data collection, some groups were over- or underrepresented. A weighting adjustment procedure was implemented considering the projections of the population distributed by sex and age groups for 2012 (2). Descriptive analysis of the final weighted sample was performed to obtain a sociodemographic description of this population.

## Results

The sociodemographic characteristics of the sample (*N* = 436) are presented in Table 3. The sample included mostly women (58.7%). The mean age was 75.2 years old (SD = 7.2 years old). The education level with higher representation was primary level (1–4 years), and a relevant percentage of the sample was illiterate (21.0%). Sixty-one percent were married/living with partner, 93.3% had children and 83.8% had grandchildren. The majority of the patients lived with a spouse or partner, with an expressive percentage living alone (15.7%). The distribution of people by urban/rural contexts was balanced (52.1% in urban areas and 47.9% in rural areas).

**Table 3:** Sociodemographic characteristics of the sample.

Sociodemographic characteristics	*N*	%
Sex	436	
Male		41.3
Female		58.7
Age	436	
Years, mean (SD)		75.2 (7.2)
Education level	434	
Illiterate		21.0
1–4 years		69.7
5–6 years		4.5
7–9 years		2.1
10–12 years		1.9
>12 years		0.8
Marital status	435	
Single		5.9
Married/lived with partner		60.9
Separated/divorced		4.5
Widowed		28.7
Children	316	
No		6.7
Yes		93.3
*n*, mean (SD)		3.4 (2.3)
Grandchildren	242	
No		16.2
Yes		83.8
*n*, mean (SD)		3.9 (3.6)
Living arrangement	432	
Alone		15.7
Partner		62.9
Children		33.6
Other relative		21.2
Other		1.1
Context	416	
Rural		47.9
Urban		52.1

**Figure 2:**
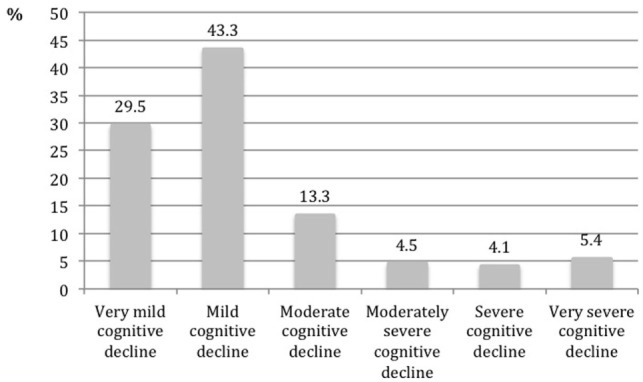
Distribution of the sample according to the stage of the GDS scale.

The distribution of the sample according to the stage of the GDS scale is presented in Figure 2. Forty-three percent of the sample was classified with mild cognitive decline, followed by 29.5% classified as very mild cognitive decline, and 13.3% as moderate cognitive decline. The most severe stages included 14% of the sample, with the stage “very severe cognitive decline” reaching 5.4%. In the group of people evaluated by the GP (*N* = 249), 39% had a formal diagnosis of dementia.

## Conclusion

The research design covering a large geographical area and the high participation of GPs in pre-screening patients from where the random sample was extracted are the main strengths of this study. The participation rate of GPs in the second phase of patients’ assessment is the major limitation. The complex methodological process to obtain data on probable dementia patients in primary care, described earlier reflects the difficulty to tackle dementia in Primary Health-Care Services. Nevertheless, this procedure may configure a pathway of care that ultimately saves time and financial resources to GPs, preventing the comprehensive assessment of older patients that are not at risk of developing dementia.

The study protocol proved to be a valuable tool for a comprehensive assessment to identify patients and characterize their health needs and staging the cognitive decline. Based on GDS, the distribution of patients by different levels of probable dementia corroborate the findings of Lopponen et al. (8) and Prince et al. (31) for developed countries.

The enrollment of the primary health-care team and of the primary caregivers in the research facilitates the access to relevant data and mobilizes attention of professionals and family to an under diagnosis and under treated disease that leaves patients and carers helplessness.

It is barely feasible or adequate to assess every old adult for cognitive decline and we know that dementia seems to be reducing its prevalence at least in UK (32). Selecting people with mental health concern before sampling appears to be a good methodological approach to arrive to a clear distribution of patients across different stages of probable dementia. This will contribute to design effective pathways of care for people with cognitive decline. Mobilizing and training GPs and other primary care professionals will foster referral of patients to customized bundles of care, leading to a global and effective plan for dementia.

## Ethics Statement

The study was submitted to the ethical committee of the ARS North—procedure number 6/2014 and approved at 7 January 2014. All the participants signed the informed consent form that was developed according the Declaration of Helsinki.

## Author Contributions

LT and CP conceived the research project design, wrote the manuscript, and conducted the data analysis. PS and AL contributed in the project conception and reviewed the manuscript. SA, MA, and MD were enrolled in the data collection and revised the manuscript.

## Conflict of Interest Statement

The authors declare that the research was conducted in the absence of any commercial or financial relationships that could be construed as a potential conflict of interest. The reviewer PB and the handling editor declared their shared affiliation.
